# Personality Profiles and Susceptibility to Digital Addiction

**DOI:** 10.1192/j.eurpsy.2026.10824

**Published:** 2026-07-31

**Authors:** N. Rmadi, R. Affes, A. Hrairi, N. Kotti, M. Hajjaji, K. Jmal Hammami

**Affiliations:** 1 Occupational medicine department, Hedi Chaker university hospital; 2 Family medicine department, Faculty of medicine of Sfax, Sfax, Tunisia

## Abstract

**Introduction:**

Individual personality profiles may predispose residents to problematic smartphone and social media use. This study examined associations between Big Five personality traits and digital addiction.

**Objectives:**

To examine the associations between Big Five personality traits and susceptibility to smartphone and social media addiction in medical residents.

**Methods:**

A cross-sectional analysis of 429 Tunisian medical residents was conducted. Personality was assessed using the BFI-20, smartphone addiction via the SAS-SV, and social media addiction with the BSMAS. Bivariate analyses compared trait scores between addicts and non-addicts.

**Results:**

Residents addicted to smartphones had significantly lower conscientiousness (2.98 ± 0.64 vs. 3.34 ± 0.60, p<0.001) and neuroticism (2.67 ± 0.51 vs. 2.88 ± 0.54, p<0.001). Similar patterns were observed for social media addiction, with lower conscientiousness (2.76 ± 0.62 vs. 3.30 ± 0.59, p<0.001) and neuroticism (2.56 ± 0.54 vs. 2.85 ± 0.51, p<0.001). Extraversion, openness, and agreeableness showed no consistent associations.

**Conclusions:**

Low conscientiousness and emotional stability appear to increase susceptibility to digital addiction among residents. These findings emphasize the need to consider personality-driven vulnerabilities when designing preventive and support interventions for young physicians.

**Disclosure of Interest:**

None Declared

